# Knowledge, attitude and practice of antibiotics: a questionnaire study among 2500 Chinese students

**DOI:** 10.1186/1472-6920-13-163

**Published:** 2013-12-09

**Authors:** Ying Huang, Jiarui Gu, Mingyu Zhang, Zheng Ren, Weidong Yang, Yang Chen, Yingmei Fu, Xiaobei Chen, Jochen WL Cals, Fengmin Zhang

**Affiliations:** 1 Department of Microbiology, Harbin Medical University, 157# Baojian Road, Nangang Disrict, Harbin, 150081, China; 2 The 2nd Affiliated Hospital of Harbin Medical University, Harbin, 150081, China; 3 Heilongjiang Province Key Laboratory for Immunity and Infection, Pathogen Biology, Harbin, 150081, China; 4 Department of General Practice, CAPHRI School of Public Health and Primary Care, Maastricht University, Maastricht, Netherlands

**Keywords:** Anbiotics usage, KAP, Questionnaire survey, Medical students, Statistical analysis

## Abstract

**Background:**

Recently, many scientists including bacteriologists have begun to focus on social aspects of antibiotic management especially the knowledge, attitude and practice (KAP) among the general population regarding antibiotic use. However, relatively few works have published on the relationship between KAP and medical education. In this study, we analyze the present status of Chinese medical (MS)- and non-medical (NS) students’ KAP on the use of antibiotics, and examine the influence of Chinese medical curriculum on the appropriate usage of antibiotics among medical students.

**Methods:**

In this study, 2500 students from 3 universities (including one medical university) in Northeastern China participate in the questionnaire survey on students’ knowledge, attitude and practice toward antibiotic usage. Wilcoxon rank sum test and Chi square test were used to analyze questionnaire-related discrete and categorical variables respectively, in order to assess the impact of the medical curriculum on students’ KAP towards antibiotics.

**Results:**

2088 (83.5%) respondents (MS-1236 and NS-852) were considered valid for analysis. The level of knowledge of MS on the proper use of antibiotics was significantly higher than that of NS (*p* < 0.0001). However, based on their responses on actual practice, MS were found to rely on antibiotics more than NS (*p* < 0.0001). Moreover, the knowledge and attitude of MS towards antibiotic use improved with the increase in grade with discriminate use of antibiotics concurrently escalating during the same period.

**Conclusions:**

This study indicates that Chinese medical curriculum significantly improves students’ knowledge on antibiotics and raises their attention on antibiotic resistance that may result from indiscriminate use of antibiotics. The study also shows an excessive use of antibiotics especially among the more senior medical students, signifying a deficiency of antibiotics usage instruction in their curriculum. This might explain why there are frequent abuses of antibiotics in both hospital and community settings from a certain angle.

## Background

In 2011, WHO set the theme of World Health Day as ‘Combat Antimicrobial Resistance: No Action Today, No Cure Tomorrow’ [1]. This shows a serious and global problem of antibiotic abuse and there is a growing consensus to urgently develop new strategies for prevention of resistance of bacteria to antibiotics. In recent years, an increasing number of researchers have focused their attention on antibiotic misuse, and follow with interest the knowledge, attitude and practice (KAP) towards antibiotics use of public [2-6].

These surveys reflect the general public’s lack of understanding on proper use of antibiotics. Thereby, it reinforces the necessity that establish certain guidelines for public education on the use of antibiotics. Such guidelines would help rationalize public practice in relation to antibiotics use [7,8]. Arguably, the public plays an important role in use (or abuse) of antibiotics as well as the dissemination of these indiscriminate tendencies [9]. Heddini *et al*., have pointed out that since antibiotics are so widely used in China, the chances of developing drug resistant microbes are much higher, and in their view, what happens in China should also be of great concern to the world [10]. Furthermore, due to the relatively relaxed regulations on antibiotics use in China, it is not difficult to obtain antibiotics without proper prescription (over-the-counter acquisition) [11,12]. Thus, the public knowledge, attitude and practice towards antibiotic use in China warrant greater attention. Besides surveys on the general public, a number of studies have also focused on different cohorts of populations including nurses of day-care centers, parents with infants, internal medicine specialists, dentists and workers in pharmacies [13-17] and others targeting medical students and interns [18-20].

At present, China antibiotic management policy has been released, but the implementation process is not strict. Doctor prescription and self-prescription co-exist. In China, the medical students major in clinical will have prescription right in the future after examination. The clinical courses offered in medical colleges generally provide students knowledge and medicine practice of biomedical bases, diagnosis, treatment, and prevention of diseases. The duration of medical and non-medical is different. Medical profession is generally 5-year, rather than non-medical profession 4-year. In the 5-year undergraduate study, students do class work in the first three years and hospital-based practice in the final two years. The clinical medicine curriculum gives some introduction of pathogenic microorganisms and infectious diseases, but does not set up antibiotics as a separate entity. Contents related to antibiotic use and resistance is mainly imbedded in chapters of pharmacology and microbiology courses. And the practice of antibiotics prescription is supervised by clinicians during students’ clinical internship.

The clinical medical students represent a highly educated group of medical personnel and their knowledge, attitude and behavior in relation to public usage of antibiotics can greatly impact in the future on antibiotic-related issues of China [10]. Distinct with the public-based researches, this study investigates medical (clinical medicine) students’ knowledge, attitude and behavior in relation to antibiotic use by controlling with non-medical students. Furthermore, this study also aims at examining the influence of Chinese clinical medicine curriculum on students’ knowledge, attitude and practice of antibiotics use.

## Methods

### Background of respondents

Respondents in this study were students from three nation-wide recruit universities in Northeastern China. All the clinical medicine students (1250) of one representative medical university of Chinese medical education participated in the study. Students from pharmacy, nursing and dentistry programs were not included. Paired 1250 non-medical respondents were randomly selected based on ID number registered in Students’ affairs office from the other two universities without clinical medicine course. All the respondents joined in with no incentives and signed the informed consents.

### Questionnaire design and grading standards

The survey was questionnaire-based and the questionnaire is designed for two objectives: to get an overview of the students’ understanding of antibiotics, and to compare the MS’ and NS’ KAP towards antibiotic use, also consider about the differences among grades. Before the main survey, a small-scale pilot study was conducted by a primary manuscript in two universities covering basic questions of antibiotics and their usage. Based on the pilot study, the questionnaire was modified and improved on advice of relevant experts from the field of statistics and epidemiology. The final version of the questionnaire had 30 questions subdivided into four categories: knowledge of antibiotics, attitude towards antibiotic use, perception of public education and practice towards antibiotic use. The questions and the grading standard can be seen in the Additional file 1.

(i) The part on knowledge of antibiotic use had 11 questions covering the normal flora of microbes, concepts of drug sensitivity and susceptibility; relationship of disease, drug resistance, and side effects of antibiotics; views on effectiveness of antibiotics among others. (ii) The part on attitude had 5 questions on the seriousness of antibiotic abuse; its influence on the student and his/her family, and the causes of the abuse. (iii) The part on the perception of perception on public education had 5 questions relating to sources of antibiotics knowledge, information channel, and eagerness to the related knowledge; college course arrangement and proper use of antibiotics campaign. (iv) The part on behavior had 9 questions ranging from the frequency of antibiotic use in fever, illnesses and various other symptoms; understanding of prescription drugs and doctor’s prescriptions; drug withdrawal status among others. When compare the overall results of each partitions all questions are concerned.

As shown in the Additional file 1, a common grading standard was used for each question in all the four categories. We graded the answers as ‘right’ or ‘wrong’ for the single-answer questions generally considering whether it corresponded with the natural situation. 2 or 0 points were assigned to the ‘right’ or ‘wrong’ answers, respectively. For the multiple-answer questions, we assigned 1 point for each ‘right’ answers and 0 points for the ‘wrong’ ones. A higher score in the first three categories presented better knowledge of antibiotics usage and more positive attitude and perceptions Also, the five-answer questions in the behavior section were graded with 1 point for ‘always’, 2 for ‘often’, 3 for ‘sometimes’, 4 for ‘seldom’ and 5 for ‘never’. A higher score here presented a lower dependency on antibiotics corresponds to better antibiotics usage behavior.

### Statistical analysis

We predefined that a questionnaire was to be considered valid for analysis if 80% of questions were answered. Wilcoxon rank sum test was used to set the grading scores (as shown above) for discrete variables (such as knowledge, attitude, public perception and behavior/practice) as well as evaluating whether statistical association exists in the three target groups; such as MS and NS, MS in different grades and NS in different grades. The statistical analyses of categorical variables (such as responses to questions on knowledge, attitude, behavior/practice and public perceptions) were done using Chi square test to assess the association between medical education and KAP towards antibiotic use. A *p* < 0.05 was considered statistically significant. Bonferroni method was employed to do multiple test correction [21]. We also apply Spearman correlation test to estimate the relations of knowledge, attitude and practice. All the analyses were done using statistics software EPIDATA3.1 and SAS9.1.

## Results

### Response and data availability

Of the 2500 questionnaires sent out, 2088 (83.5%) were considered valid (with 80% of questions in questionnaire answered) and suitable for analysis. Of these, 1236 first- to fifth-year medical students constituting the main target group, while 852 first- to fourth-year non-medical students were used mainly as control (Table 1).

**Table 1:** Distribution of 2088 respondents from different universities and grades

**Grade**	**MS**	**NS**	**Total**
Yr 1	262	141	403
Yr 2	244	323	567
Yr 3	216	223	439
Yr4	273	165	438
Yr 5	241	\	241
Total	1236	852	2088

### The influence of medical curriculum on MS’ knowledge on antibiotic use

The results show that overall MS scored remarkably better than NS on knowledge of antibiotic use (*χ*^*2*^ = 191.8869, *p* < 0.0001) while no significant difference was found between first-year MS and first-year NS (*χ*^*2*^ =1.2190*, p* = 0.2696). The same pattern of results was observed when answers of each of the questions among MS and NS were analyzed using *χ*^*2*^ test (Table 2). For example, on the question of effectiveness of antibiotics on treating bacterial infection, 92.93% of MS provided the right answer compared to 81.81% for NS (*p* < 0.0001). Moreover, 64.52% of the MS chose ‘antibiotics cannot treat viral infection’, compared to only 43.44% among the NS (*p* < 0.0001). At the first-year level there was no striking differences between MS and NS in their knowledge of antibiotics use. However, final year MS had significantly higher scores compared to first-year MS with MS’ knowledge improving more appreciably than NS throughout the program (Table 2).

**Table 2:** Students’ knowledge on antibiotic use

**Question (correct response)**	**Total % (n/N)**	**Whole % (n/N)**				**1st year % (n/N)**				**Final year % (n/N)**			
		**MS**	**NS**	***χ***^***2***^	***p***	**MS**	**NS**	***χ***^***2***^	***p***	**MS**	**NS**	***χ***^***2***^	***p***
Can antibiotics cure bacterial infections? (yes)	88.4 (1818/2057)	92.9 (1130/1216)	81.8 (688/841)	59.87	<0.0001*#	88.2 (231/262)	85.7 (120/140)	0.50	0.4813	93.5 (215/230)	79.6 (129/162)	16.96	<0.0001*
Can antibiotic cure viral infections? (no)	55.9 (1146/2050)	64.5 (782/1212)	43.4 (364/838)	89.35	<0.0001*#	49.7 (130/262)	48.2 (67/139)	0.07	0.7871	69.1 (159/230)	39.1 (63/161)	34.73	<0.0001*
Do you think the use of antibiotics will speed up the recovery of cold, cough? (no)	25.7 (531/2064)	27.4 (333/1214)	23.3 (198/850)	4.48	0.0344*	23.1 (60/260)	19.2 (27/141)	0.83	0.3622	31.0 (72/232)	20.7 (34/164)	5.20	0.0226*
Have you heard of antibiotics resistance? (yes)	91.4 (1873/2050)	94.7 (1146/1210)	86.6 (727/840)	41.88	<0.0001*#	89.7 (235/262)	93.6 (132/141)	1.73	0.1879	89.2 (215/241)	78.2 (129/165)	9.21	0.0024*
Do you think frequent use of antibiotics will decrease efficacy of treatment when using the antibiotic again? (yes)	84.9 (1744/2055)	87.9 (1063/1210)	80.6 (681/845)	20.42	<0.0001*#	85.8 (223/260)	83.7 (118/141)	0.31	0.5769	85.2 (196/230)	76.4 (123/161)	4.90	0.0268*
Is the efficacy better if the antibiotics are newer and more costly ? (no)	77.7 (1606/2066)	81.5 (993/1219)	72.4 (613/847)	23.84	<0.0001*#	72.5 (190/262)	72.9 (102/140)	0.01	0.9423	79.3 (184/232)	70.7 (116/164)	3.85	0.0497*

*p <0.05; #Significance after multiple test correction.

### The influence of medical curriculum on MS' attitude and perception of public education

The results showed that on the questions of attitude on antibiotic use, the MS scored significantly higher than the NS (*χ*^*2*^ = 90.7200*, p* < 0.0001). A similar pattern was obtained from the analyses of the students’ perception of public education (*χ*^*2*^ = 40.5875*, p* < 0.0001). However, when the perception of only first-year students was compared, there was no significant difference between MS and NS in relation to their awareness of public education on antibiotic use (*χ*^*2*^ = 0.6291*, p* = 0.4277). Analysis of responses to each question, found that 82.84% of the MS believed that drug resistance in bacteria has become a problem in China, and this was much higher than that of NS (62.80%) (*p* < 0.0001). Furthermore, 83.88% of the MS considered abuse of antibiotics was the main cause of drug resistance, while only 61.75% of the NS did so (*p* < 0.0001)*.* In addition, 81.96% of the MS and 74.14% of the NS thought it was necessary to launch certain large scale publicity to promote understanding of antibiotics (*p <* 0.0001, Table 3).

**Table 3:** Students’ attitude and public education on usage of antibiotics

**Question (agree)**	**Total % (n/N)**	**Whole % (n/N)**				**1st year % (n/N)**				**Final year % (n/N)**			
		**MS**	**NS**	***χ***^***2***^	***p***	**MS**	**NS**	***χ***^***2***^	***p***	**MS**	**NS**	***χ***^***2***^	***p***
There is abuse on antibiotics at present. (agree)	86.1 (1739/2019)	90.1 (1069/1187)	80.5 (670/832)	37.19	<0.0001*#	87.2 (225/258)	76.4 (107/140)	7.63	0.0058*	91.7 (200/218)	77.9 (123/158)	14.61	<0.0001*
Antibiotics resistance has become a problem in China. (agree)	74.6 (1534/2056)	82.8 (1004/1212)	62.8 (530/844)	105.50	<0.0001*#	67.2 (176/262)	52.5 (74/141)	8.40	0.0037*	82.6 (199/241)	64.8 (107/165)	16.57	<0.0001*
Abuse of antibiotics has become the main cause leading to bacterial resistance. (agree)	74.5 (1533/2059)	83.9 (1010/1212)	61.8 (523/847)	122.14	<0.0001*#	72.9 (191/262)	65.0 (91/140)	2.72	0.0991	81.4 (188/231)	55.0 (89/162)	32.01	<0.0001*
Antibiotic resistance affects you and your family’s health. (agree)	76.6 (1578/2059)	81.9 (993/1213)	69.2 (585/846)	45.00	<0.0001*#	69.7 (182/261)	70.7 (99/140)	0.04	0.8377	84.4 (195/231)	63.0 (102/162)	23.74	<0.0001*
Necessary to get more education about antibiotics. (agree)	85.1 (1751/2058)	89.2 (1082/1213)	79.2 (669/845)	39.47	<0.0001*#	89.3 (234/262)	80.0 (112/140)	6.60	0.0102*	87.0 (201/231)	79.5 (128/161)	3.97	0.0464*
Need to establish course on ‘rational use of antibiotics’ at university. (agree)	67.3 (1388/2062)	74.2 (901/1214)	57.4 (487/848)	63.95	<0.0001*#	61.1 (160/262)	61.0 (86/141)	0.00	0.9881	74.5 (172/231)	56.4 (92/163)	14.03	0.0002*
Necessary to carry out large-scale ‘antibiotics campaign’ promotion. (agree)	78.8 (1623/2061)	82.0 (995/1214)	74.1 (628/847)	18.21	<0.0001*#	77.5 (203/262)	74.5 (105/141)	0.46	0.4968	82.3 (190/231)	75.3 (122/162)	2.80	0.094

*p <0.05; #Significance after multiple test correction.

### The influence of medical curriculum on the MS’ behavior towards antibiotics use

It is interesting to note that our data revealed that MS scored more poorly than the NS on the behavior towards the use of antibiotics. (*χ*^*2*^ = 101.2526*, p* < 0.0001). However, there was no significant difference observed when data from only first-year students from both groups were compared (*χ*^*2*^ = 0.0057*, p* = 0.9399). Moreover, 49.84% of MS versus 40.02% of NS advocated use of antibiotics on patients with low fever (< 38.5°C) (*p* < 0.0001) while 13.59% of MS would use antibiotics with high frequency on common cold compared to 8.45% of NS (*p* = 0.0003). Furthermore, 19.15% of the MS would actively request doctors to prescribe antibiotics compared with only 12.44% NS (*p* = 0.0002). When symptoms of respiratory tract infection occur, MS would advocate use of antibiotics more frequently than NS (Table 4). In addition, the proportion of MS (75.29%) who had frequently used antibiotics without the doctor’s prescription previous occasions was much higher than that of NS (49.46%) (*p* < 0.0001).

**Table 4:** Students’ behavior of using antibiotic

**Question (response)**	**Total % (n/N)**	**Whole % (n/N)**				**1st year % (n/N)**				**Final year % (n/N)**			
		**MS**	**NS**	***χ***^***2***^	***p***	**MS**	**NS**	***χ***^***2***^	***p***	**MS**	**NS**	***χ***^***2***^	***p***
Use antibiotics when having fever (temperature lower than 38.5°)	45.8 (957/2088)	49.8 (616/1236)	40.0 (341/852)	19.57	<0.0001*#	37.8 (99/262)	39.7 (56/141)	0.14	0.7041	55.6 (134/241)	38.8 (64/165)	11.08	0.0009*
Common cold (always, often)	11.5 (240/2088)	13.6 (168/1236)	8.5 (72/852)	13.11	0.0003*#	10.3 (27/262)	6.4 (9/141)	1.73	0.1879	14.5 (35/241)	9.7 (16/165)	2.08	0.1496
Acute bronchitis (always, often)	22.9 (479/2088)	29.5 (364/1236)	13.5 (115/852)	72.60	<0.0001*#	17.6 (46/262)	14.2 (20/141)	0.76	0.3829	39.8 (96/241)	15.8 (26/165)	27.01	<0.0001*
Coughing up yellow/green sputum (always, often)	27.6 (576/2088)	37.9 (468/1236)	12.7 (108/852)	160.18	<0.0001*#	18.3 (48/262)	14.9 (21/141)	0.76	0.3837	56.4 (136/241)	12.1 (20/165)	81.28	<0.0001*
Sore throat (always, often)	15.3 (320/2088)	18.9 (233/1236)	10.2 (87/852)	29.01	<0.0001*#	14.9 (39/262)	10.6 (15/141)	1.43	0.2326	20.8 (50/241)	10.9 (18/165)	6.80	0.0091*
Cough with fever (always, often)	19.9 (415/2088)	24.5 (303/1236)	13.2 (112/852)	40.93	<0.0001*#	11.5 (30/262)	12.1 (17/141)	0.03	0.8565	28.6 (69/241)	7.3 (12/165)	27.98	<0.0001*
Congested nose with headache (always, often)	12.5 (261/2088)	15.0 (185/1236)	8.9 (76/852)	16.86	<0.0001*#	9.9 (26/262)	10.6 (15/141)	0.05	0.8209	15.8 (38/241)	5.5 (9/165)	10.18	0.0014*
Coughing up white sputum (always, often)	9.4 (196/2088)	10.9 (135/1236)	7.2 (61/852)	8.39	0.0038*	4.6 (12/262)	6.4 (9/141)	0.60	0.4374	17.8 (43/241)	9.7 (16/165)	5.23	0.0222*
Cough lasting 2 weeks or more (always, often)	27.6 (576/2088)	32.6 (403/1236)	20.3 (173/852)	38.20	<0.0001*#	23.7 (62/262)	20.6 (29/141)	0.50	0.4782	35.7 (86/241)	18.2 (30/165)	14.70	0.0001*
Asked doctors to prescribe antibiotics when you catch a common cold (yes)	16.4 (335/2042)	19.2 (231/1206)	12.4 (104/836)	16.99	0.0002*#	9.6 (25/261)	8.6 (12/140)	0.11	0.7397	29.8 (68/228)	11.5 (18/157)	18.07	<0.0001*

*p <0.05; #Significance after multiple test correction.

### Comparison of responses from different grades (academic year) of the medical students

**Figure 1:**
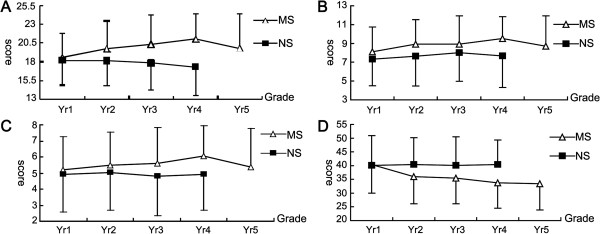
**The grading graphs of students’ antibiotics knowledge, attitude, practice and views on promotion of understanding of antibiotics. A**. Grading graph of students’ knowledge in relation to antibiotics. **B**. Granding graph of students’ attitude in relation to antibiotics. **C**. Granding graph of students’ public perception in relation to antibiotics. **D**. Granding graph of students’ practice in relation to antibiotics

In order to further explore the influence of the medical curriculum on students’ knowledge, attitude and behavior or practice regarding usage of antibiotics at different stages of medical education, we analyzed and compared the responses of MS from different grades with that of NS. The results showed that the answers varied significantly among the MS in different grades. The students’ knowledge improved as they progressed in their study attaining the highest scores at third- and fourth-years with a slight decrease in the fifth-year (Figure 1A), suggesting that medical curriculum enhanced their understanding of antibiotics use. Similarly, their attitude on the use of antibiotics was only slightly improved (year 4) over the same period with a clearer recognition of antibiotic abuses and development of drug resistance in bacteria (Figure 1B). Figure 1C shows that the demand for public education to promote the awareness for proper use of antibiotics increased and reaching its peak in 4th year, again with a slight slip-back in the final year. The most surprising, however, was the finding that the MS’ score was lower on behavior/practice towards antibiotic use as compared to NS (Figure 1D). There was no significant difference among non-medical students of all grades in terms of their knowledge, attitude and practice towards antibiotics.

## Discussion

This study surveyed MS and NS from three universities in the Northeastern China to assess their knowledge, attitude and practice towards antibiotic use in the form of questionnaires. MS were better than NS in terms of knowledge, attitude and perception on the level of public education on antibiotic use, but worse on behavior. Our study also reveal that the senior MS has a more positive behavior on the usage of antibiotics compared to lower grade MS and NS in general, despite MS would expose to antibiotics education and understand the serious consequences of antibiotic abuse during their earlier years in the medical school.

The result of knowledge studies show that medical students can get more information about antibiotics than other students or public, The increase of knowledge can be well presented by the relative specialized question whether antibiotics can cure viral infection which is described in result partition, whenever it is compared between MS and NS and between grades of MS (69% of year 5 and 49% of year 1). Some similarity can be drawn from the study in Jordan in 2012 by Suaifan *et al.* and in 2011 by Shehadeh *et al.* that highlights existing gaps in the knowledge of MS and NS on antibiotics use [22,23]. The difference, however, lies in the fact that in Jordan self-medication as a result of lack of knowledge on antibiotics plays a leading role in the misuse antibiotics while in China it is the clinicians with the right to prescribe that are at the forefront of antibiotic abuse.

Our study result also prompt that the antibiotic knowledge and attitude education should be strengthened in China. More many MS believed that antibiotics can speed up recovery of common cold, cough and a number of other related illnesses arising from viral infections. This is previously an important cause of antibiotic over-use. 74% of the MS in our study favored the establishment of a course on rational use of antibiotics at university level in China to boost their knowledge level (Table 3). Minen *et al.* in a KAP survey on antibiotics among 304 MS in America 2010, reported that more than 75% of the students preferred more education on antibiotics which is consistent with our study (89%) [18]. According to a research among 503 interns and senior physicians in France, 98% of physicians considered antibiotic resistance as a national problem, whereas only 83% of the Chinese medical students shared this view[19] underscoring the great need for sensitization in China.

When we carried about the relationship of knowledge, attitude and behavior, our correlation analysis showed that students’ knowledge on antibiotics had a positive correlation with their attitude and awareness of importance of public education, indicating that there was a direct relationship between their knowledge level and attitude towards promotion of public awareness (Spearman correlation coefficient R = 0.3474, *p* < 0.0001). Our data indicate that there was no direct correlation between the student’s knowledge, attitude or public perception and their behavior towards antibiotics use. Today, many countries like Britain and Holland are focusing on public education aimed at changing the irrational and indiscriminate use of antibiotics in the community in order to delaying curtail the development resistance to antibiotics [9,24]. Whereas some researchers have reported that public education alone may not necessarily improve the tendency misuse and abuse antibiotics in the society [3], other works argue that providing clear guidelines for medical practitioners may provide a quicker and more effective route to prudent and rational use of antibiotics eventually reversing the current trend [25,26].

We analyze from the level of different grades to research the KAP towards antibiotics use, which is an important feature of our study. Figure 1A, B, C and D show the comparisons between MS’ and NS’ levels of knowledge, attitude, perception of public education and behavior towards antibiotic use. The MS’ knowledge, attitude and perception on antibiotic use improved as they progressed in course peaking at year 4 with a slight scale-back at the year 5. It is worth noting that the clinical medicine students are introduced antibiotics and antibiotic-related information from the second year in courses such as microbiology (year 2), pharmacology (year 2), internal medicine (year 3), and epidemiology (year 4). Although they could touch prescription of antibiotics during the years 4 and 5, MS do not prescribe any medicines until after they have completed the course. The most senior (years 4 and 5) MS’ scores on behavior towards antibiotics use were lower than those of their junior colleagues (years 1, 2 and 3) indicating that there could be some negative impact on students’ erstwhile good antibiotic practices by the hospital environment during clinical. This fluctuation of KAP could be due to inherent flaws in the medical curriculum. And it reflect that add some courses on rational antibiotic practice in the medical curriculum in China is urgently needed.

This is a large sample size (2500) and high response rate (83.5%) research on KAP towards antibiotics usage of MS and NS. And we compare year groups for both MS and NS from representive universities which would reflect the national situation of China, to some extent. On this basis, we propose that it should extra focus on the antibiotic usage and prescription practice introduction of MS in the medical curriculum, and at the same time strengthen the antibiotic knowledge education for MS and public. However, we suggest that future surveys of this nature may endeavor to cover wider geographical regions of the country.

## Conclusions

This survey of 2500 MS and NS reveals that the Chinese medical curriculum significantly improves students’ knowledge on antibiotics and antibiotic-related issues. The study also shows an excessive use of antibiotics especially among more senior medical students, reflecting a deficiency of antibiotics usage instruction in their curriculum. This survey also truly reflects some of potential causes of antibiotic abuse in the typical Chinese hospital. To curb the proliferation antibiotic misuse and its consequent effects, we propose the establishment of a special course on rational prescription of antibiotics that emphasizes more on the behavior of medical students towards antibiotics use rather than advance of knowledge alone.

### Ethical approval

All the participants signed the informed consent.

## Competing interests

All authors declare that they have no competing interest.

## Authors’ contributions

FZ, YH, JG and YF conceived of the study and participate in its design. MZ, ZR and WY did the questionnaire survey and collected the data. YH, YC and XC performed the statistical analysis and interpretation of the data. YH, YC and FZ drafted the manuscript. JC revised the manuscript critically for important intellectual content. All authors read and approved the final manuscript.

## Pre-publication history

The pre-publication history for this paper can be accessed here:

http://www.biomedcentral.com/1472-6920/13/163/prepub

## Supplementary Material

Additional file 1 Questions and the grading standard of the questionnaire. Click here for file
